# Pomegranate peel extract ameliorates the severity of experimental autoimmune encephalomyelitis via modulation of gut microbiota

**DOI:** 10.1080/19490976.2020.1857515

**Published:** 2020-12-17

**Authors:** Xin-Yu Lu, Bing Han, Xin Deng, Si-Ying Deng, Yan-Yan Zhang, Pei-Xin Shen, Teng Hui, Rui-Heng Chen, Xing Li, Yuan Zhang

**Affiliations:** a National Engineering Laboratory for Resource Development of Endangered Crude Drugs in Northwest China, The Key Laboratory of Medicinal Resources and Natural Pharmaceutical Chemistry, The Ministry of Education, Shaanxi Normal University, Xi’an, Shaanxi, China; b Department of Medical Technology, Xi’an Medical University, Xi’an, Shaanxi, China; c The High School Affiliated to Shaanxi Normal University, Xi’an, Shaanxi, China

**Keywords:** multiple sclerosis, experimental autoimmune encephalomyelitis, pomegranate peel extract, gut microbiota, 16s rRNA gene sequencing

## Abstract

Multiple sclerosis (MS) is a CNS autoimmune disease characterized by demyelination and inflammatory infiltration with a high disability rate. Increasing evidence has demonstrated the importance of gut microbiota as an environmental risk factor in MS and its animal model experimental autoimmune encephalomyelitis (EAE). Diet is the main determinant of gut microbiota composition and function, which greatly affects the shaping of microbial structure. Pomegranate peel, a waste product in the production of juice, is rich in health-promoting compounds. However, its individual constituents, immunoregulatory activities, and action associated with bacterial diversity in the gut microbiota are largely unknown. Here, the main nutrient ingredients of pomegranate peel extract (PPE) were identified as phenols, flavonoids, amino acids, carbohydrates, fatty acids, lipids, nucleotides, organic acids, alcohols, and vitamins via metabolomics evaluation. We found, for the first time, oral PPE (100 mg/kg/day) not only effectively relieves EAE, inhibits CNS inflammatory factor infiltration and myelin loss, but also reshapes gut microbiota. Furthermore, recipient EAE mice with fecal transplantation from the PPE-treated donor delayed the disease development significantly. 16S rRNA gene sequencing revealed the increased gut microbiota richness in PPE-treated group. Among them, Lactobacillaceae enriched significantly, while Alcaligenaceae and Acidaminococcacea decreased remarkably. In conclusion, our data demonstrated that gut microbiota mediated the beneficial effects of oral PPE on EAE, and provided new ideas for developing the prebiotic value of pomegranate peel for the treatment of autoimmune diseases.

## Introduction

Multiple sclerosis (MS), an inflammatory demyelinating disease affecting the central nervous system (CNS), has multiple clinical manifestations including vision loss, exercise, sensory impairment, or disability.^1^ It is generally believed that MS is caused by peripherally activated autoreactive effector factor CD4^+^ T cells that cross the blood–brain barrier (BBB) into the CNS, where they are re-activated by antigen-presenting cells (APCs) and secrete inflammatory cytokines (e.g., IFN-γ, TNFα, IL-17, GM-CSF, and IL-22). Simultaneously, these cytokines activate CNS-resident immune cells (microglia, astrocytes, and macrophages) leading to axonal or neuronal degeneration, demyelination, astrocytic gliosis, as well as breakdown of the blood–brain barrier.^2,3^ The cause of MS is elusive and multifaceted with associations to genetic susceptibility, infection, inflammation, and environmental factors. Recently, based on experimental autoimmune encephalomyelitis (EAE), one of the most widely studied animal models of MS, it has been found that gut microbes have become a novel and critical environmental risk factor for the development of MS.^4^

Approximately 100 trillion microbes inhabit the intestines of organism, interacting with the intestinal immune system to affect intestinal development and function. It is known as an imbalance of the gut microbiota results in numerous diseases, including Alzheimer’s disease, obesity, diabetes mellitus, inflammatory bowel disease (IBD), and cancer.^5,6^ Recently studies show that the gut microbiota plays a role in the pathogenesis of MS. Germ-free animals have significantly reduced neuroimmune disease symptoms and pathology compared to mice colonized with microbes while expressing lower levels of inflammatory cytokines and higher levels of Foxp3^+^ Treg cells associated with EAE.^7^ Kerstin Berer et al. reported that transplanted fecal samples from MS-affected twins into germ-free mice enable spontaneous autoimmune encephalomyelitis in mice.^8^ Another study demonstrated that oral *L. murinus* ameliorates salt-induced aggravation of actively induced MOG_35-55_ EAE.^9^ Dietary habits rapidly alter the composition of gut microbiota, such as intermittent fasting (IF), improves the clinical course of EAE, and changes the gut bacteria richness.^10^ Treatment with short-chain fatty acids (SCFAs) ameliorated EAE and reduced axonal damage, suggesting that SCFAs may have a beneficial anti-inflammatory effect in MS. Conversely, by increasing the pathogenic Th1 and/or Th17 cell in the small intestine, treatment with long-chain fatty acids (LCFAs) reduced short-chain fatty acids in the gut and aggravated the disease.^11^ Therefore, it will be necessary to explore how to establish a desirable gut microbiome through dietary regulation to prevent or treat the occurrence of MS.

Pomegranate (*Punica gran*atum L., Punicaceae), is a popular fruit that has been awarded the title of “super food” due to its immense health benefits.^12^ Pomegranate extracts (PEs) contain bioactive molecules that are reported to be antibacterial, antiviral, hypolipidemic,^13^ and anti-inflammatory. Roughly 10% seeds and 40% aril make up the edible part of pomegranate, while the inedible peel accounts for about 50% of the whole fruit.^14^ At present, the pomegranate processing industry mainly focuses on the development of the edible part, such as the production of new beverages with health care and therapeutic functions. Peel is rich in hundreds of bioactive phytochemicals (e.g., polyphenols, anthocyanins, and hydrolyzable tannins)^15^ but is often discarded as waste in daily life. Modern pharmacological research suggests that pomegranate peel has multiple bioactivities, such as anti-oxidation, anti-inflammatory,^16^ and immunomodulatory,^17^ indicating its potential for the treatment of autoimmune diseases or chronic inflammation. Generally, phytochemicals are characterized by low bioavailability. Some studies suggest that 90–95% of the unmodified phenolic compounds accumulate in the large intestine, where they are broken down by the gut microbes to supply other biologically active substances.^12,18^ Valeria Sorrenti *et al*. reported the additive beneficial effects of pomegranate peel extracts (PPE) combined with LGG-T1 probiotic supplementation (*L. rhamnosus* GG ATCC 53103 strain) on both preventing lipid accumulation in obesity, and reducing tissue inflammation,^13^ suggesting that the health-promoting potential of PPE and probiotics on pre-adipocyte differentiation. However, whether PPE can exert a therapeutic effect on EAE by regulating gut microbiota is unclear.

In this study, the chemical composition of PPE was tested, and the therapeutic effects and mechanism of PPE on autoimmune diseases were evaluated in the EAE model. Our data suggested that PPE serves as a potential dietary supplement prebiotic, which benefits for autoimmune diseases by improving the gut microbiota structure.

## Materials and methods

### EAE induction and animal diet

Female C57BL/6 mice (~8 weeks) were purchased from SPF (Beijing) Biotechnology CO., ltd. Mice were housed at the in-house animal care facility of Shaanxi Normal University animal under a 12-hour day-night-cycle and standardized conditions (23°C ± 2°C). All experiments were approved by the Institutional Animal Care and Use committee of Shaanxi Normal University and were in accordance with the approved institutional guidelines and regulations. EAE was induced according to our previous study.^19,20^ EAE mice were randomly divided into two treatment groups, 100 mg/kg PPE or solvent (PBS) were applied daily via oral gavage, at the first day of immunization, onset of disease (10 d), and peak of disease (16 d). After immunization, mice were assessed clinically in a blinded manner using a disease severity scale that is scored according to a 0–5 scale as described previously.^20^ Briefly, 0, no clinical symptoms; 0.5, stiff tail; 1, limp tail; 1.5, limp tail and stagger gait; 2, paralysis of one limb; 2.5, one limb was paralyzed and the other was faint; 3, both hind limbs were completely paralyzed; 4, moribund; and 5, death.

### Fecal microbiota transplantation

For transplantation, collection of fecal matter from mice maintained on PPE or solvent (PBS) (donor mice) in a sterile manner. Fecal microbiota transplantation (FMT) is based on established methods. Briefly, stools from donor mice of each diet group were pooled and 100 mg was resuspended in 1 mL of sterile water. The solution was vigorously mixed using a table vortex for 10 s, before centrifugation at 800 *g* for 3 min. Eight-week-old female recipient mice (n = 5 for each transplant group) were immunized with MOG_35-55_ to induce EAE and FMT was performed daily after immunization. Fresh supernatant (100 μL for each mouse) was prepared on a daily by oral gavage for each recipient mouse, and clinical scores were observed.

### Plant material and extraction

Pomegranate fruits were obtained from Xi’an Lin Tong of Shaanxi Province (China).

Peels were separated from fruit manually and dried in an oven at 30–40°C. Dry samples were grounded with a laboratory grinder. Powdered pomegranate peel (0.5 g) was extracted with 15 mL of 60% ethanol at room temperature (25°C) for 12 h, and then ultrasonic for 30 min. After filtration and combination, the extract was evaporated using a rotary evaporator. Finally, the aqueous fraction was freeze-dried to get brown PPE powder solid, which was kept at 4°C in the dark for further examination.

### Determination of tannins of PPE

Total tannin content was calculated using the method described in the pharmacopeia of the People’s Republic of China (2015), with minor modifications. Briefly, for assaying total phenolics, prepare a 2 mg/mL extract sample with distilled water. In order to determine polyphenols that are not adsorbed, pipette 5 mL extract sample (2 mg/mL) solvent was mixed with 120 mg Caseins (Solarbio, China) for 1 h at 30°C water bath, with shake constantly, filter for next testing. The above test samples were treated with 500 μL of phosphomolybdotungstic reagent (Phygene, China). After the addition of 5 mL water and 6 mL 29% Na_2_CO_3_, mixed thoroughly, the reaction tube was incubated at room temperature for 30 min, finally, the absorbance was read at 760 nm versus a blank. Gallic acid was used as a standard for the calibration curve, and the calibration equation was *y*= 2.2406*x*+0.1496, *R*^2^ = 0.998. Quantitation of tannins was performed using the following equation: tannins = total phenolics-polyphenols that are not adsorbed. Measurements were performed in triplicate.

Ellagic acid is an indicator component of pomegranate peel recorded in the Pharmacopoeia of the People’s Republic of China (2015). Qualitative and quantitative determination of ellagic acid (EA) in PPE by high-performance liquid chromatography (HPLC, Therom UltiMate3000). PPE extract sample was dissolved using methyl alcohol (Fisher). Chromatographic separation on a Waters Symmetry C18 column (5 μm, 4.6 × 250 mm). The mobile phase is composed of 0.2% phosphoric acid (A) and methanol (B) in a ratio of A:B = 45:55. The flow rate, column temperature and injection volume were set to 1.0 mL/min, 30°C, and 5μL respectively. The chromatogram was measured at 256 nm. Identify the main peak of the sample by comparison with EA standards (DESITE, China), calculation of EA content in PPE samples based on EA standard curve (*y*= 756.77*x*+0.2343, *R^2^ *= 0.9907).

### GC-MS analysis of PPE

GC-MS system (GC: Agilent 7890B; MS: Leco Pegasus HT) was performed for structural characterization on a DB-5 MS column (30 m×0.25 mm×0.25 μm). The PPE extract was dissolved in methanol to 10 mg/mL, centrifuged for 10 min (13200 rpm, 4°C), and 100 μL of the supernatant was added to the injection bottle. After concentration and drying, 45 μL of methoxyamine (20 mg/mL dissolved in pyridine) was added, with shaking at 37°C for 1 h. Then, add 46 μL of MSTFA (containing 3.33% RI), shaking at 37°C for 2 h, allowing to stand for 1 h before being used for GC-MS analysis. Helium was used as a carrier and the flow rate was maintained at 1 mL/min. 1 μL of sample was injected into the column. The oven initial temperature was set to 80°C for 2 min, increase to 300°C at a rate of 12°C/min holding with a 5.7 min period. The temperatures of the transfer line and ion source were maintained at 270°C and 220°C, respectively. Identification and quantification of the components were performed using the database of the National Institute of Standards and Technology (NIST 05 and NIST 05s).

### Fecal DNA extraction and sequencing analysis of gut microbiota

Mice fecal samples were collected and stored at −80°C until DNA extraction. DNA was extracted using a commercial TIANamp Stool DNA Kit (TIANGEN, Beijing) according to the manufacturer’s instructions. Evaluation of DNA concentration and purity using a NanoDrop spectrophotometer (Thermo Fisher Scientific). Detection of extracted genomic DNA by 1% agarose gel electrophoresis. The V3-V4 hypervariable region of the bacterial 16S rRNA gene was amplified using the primers 338 F: 5ʹ-ACTCCTACGGGAGGCAGCAG-3ʹ and 806 R: 5ʹ-GGACTACHVGGGTWTCTAAT-3ʹ. PCR products were quantified using the QuantiFluor™-ST (Promega, USA) fluorescence system. DNA libraries were constructed (TruSeqTM DNA Sample Prep Kit) and sequenced using the Illumina MiSeq system.

### Bioinformatics analysis

MiSeq sequencing results in double-ended sequence data, PE reads were spliced according to the overlap relationship, quality control and filtering were performed on the sequence quality. USEARCH (version7.0 http://drive5.com/usearch/) was used to cluster the operational taxonomic units (OTUs) with 97% similarity of the sequences, removing the chimeric sequences. Using the RDP Classifier (version2.2 http://sourceforge.net/projects/rdpclassifier/) with a threshold of 70%, to count the Community compositions at different taxonomic levels (domain, kingdom, phylum, class, order, family, genus, species).

## Results

### Metabolite profiling of PPE

Our metabolomics evaluation of PPE involved quantification of total phenols and tannin by spectrophotometry, ellagic acid-targeted analyses by HPLC (Supplementary Figure S1(a, b)), and non-targeted analysis by GC/MS (Supplementary Figure S1(c, d)). The content of total phenols and tannin in PPE was 225.90 ± 2.96, 192.68 ± 3.06 mg/g, respectively. Identification by comparison of UV spectra and retention times with standard, our data showed that the ellagic acid content in the PPE was 5.97 ± 0.008 mg/g. Next, the composition of PPE was investigated using GC-MS. The total ion chromatogram (TIC) of this assay was shown in Supplementary Figure S1(c). These compounds included amino acids (L-Malic acid, L-Aspartic acid), carbohydrates (L-Arabinose, L-Arabitol, D-Fructose, D-Mannose, L-Sorbose, D-Galactose, Mannitol), organic acids (L-Lactic acid, Oxalic acid, Shikimic acid), Alcohols (Glycerol), Fatty Acids (Palmitic acid), and Flavonoids (Genistein) (Supplementary Figure S1(d)). Among them, L-Sorbose has the highest relative content (30.29%). Details of the chemical composition of PPE were shown in Supplementary Table S1.

### Oral PPE treatment remarkably alleviated EAE clinical course

**Figure 1.**
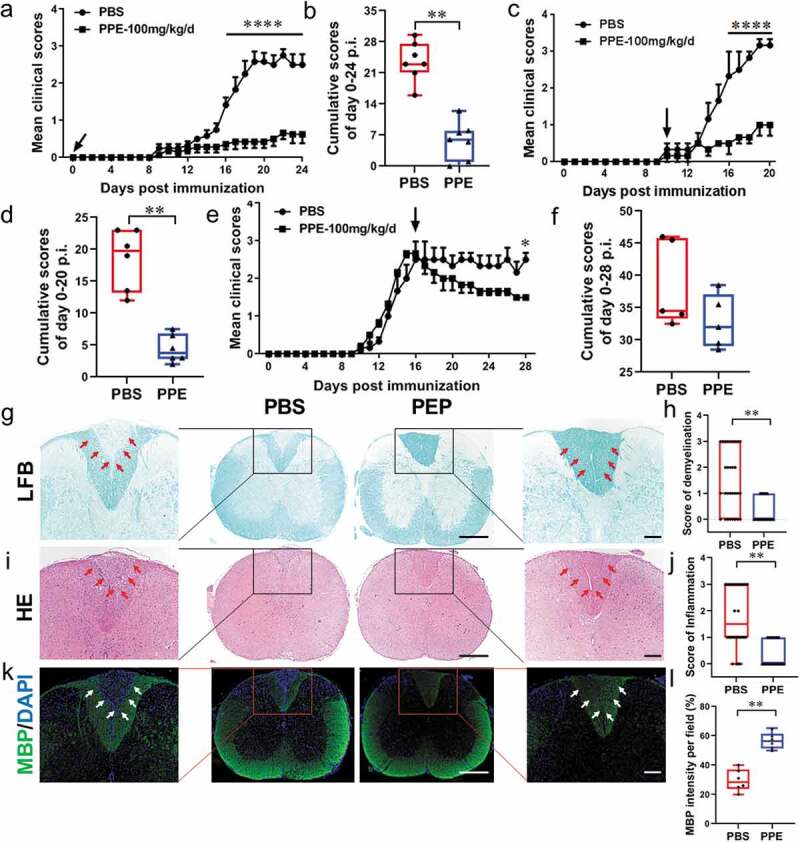
Oral PPE treatment remarkably alleviated EAE. C57BL/6 mice were immunized and treated by oral gavage with PBS or PPE (100 mg/kg/day) at d 0 post-immunization (p.i.) (a, n = 7), d 10 p.i. (c, n = 6), and d 16 p.i. (e, n = 5) Arrow indicated treatment time point. (b, d, f) Cumulative scores were the statistical analysis of the total of daily clinical scores from a, c, and e. PBS- or PPE -treated EAE mice described in (a) were sacrificed at d 24 p.i., and spinal cords were isolated for immunohistochemical staining. (g, h) LFB staining and statistical analysis. (i, j) H&E staining and statistical analysis. (k, l) MBP immunofluorescence staining and intensity quantization. Full slice bar = 500 μm, partial slice bar = 200 μm. Data are expressed as mean ± SEM, *p < 0.05, **p < 0.01, ***p < 0.001, and ****p < 0.0001, determined by two-way ANOVA (a, c, e), or unpaired Student’s *t*-test (b, d, f, h, j, l). Data are represented by three independent experiments

To investigate whether oral administration of PPE treats neuroinflammation and/or inflammatory autoimmune diseases, we used an MOG_35–55_-induced EAE mouse model, a classic model that recapitulates human MS. The effect of PPE administration on disease prevention (oral treatment starting from the day of MOG_35–55_ immunization) was examined. Daily PPE treatment significantly reduced EAE clinical scores and severity compared with the PBS-treated group (Figure 1(a, b)). Myelin destruction and complete axonal dysfunction in the CNS were the main pathologic hallmark of MS.^21^ Separation of the spinal cord for histological analysis to evaluate pathological changes, mice in the PPE group had less inflammatory infiltration and demyelination compared to the control group (*p* < 0.01, Figure 1(g–j)). The MBP density in the spinal cord sections of the mice was examined to further confirm the extent of demyelination. The results showed that mice with oral PPE had higher MBP density and smaller demyelinated areas (*p* < 0.01, Figure 1(k, l)).

Further testing for the benefcial effects of oral PPE on ongoing EAE was performed, in which the mice were fed PPE daily on d 10 (onset) or on d 16 (peak) of the EAE clinical course. As shown in Figure 1(c), the maximum clinical score of the PBS group was 3.16 ± 0.29 at 19 d after EAE induction, while PPE-treated mice showed a maximum EAE score of 1.00 ± 0.50 with the significantly lower cumulative scores than that if controls (*p* < 0.05, Figure 1(d)). PPE treatment, at both time points, effectively reduced disease severity and inhibited EAE progression (Figure 1(c–f)). Overall, these data demonstrated that PPE has a significant effect on reducing EAE clinical severity and CNS pathology.

### Oral PPE suppressed CNS infiltration and inflammation

**Figure 2.**
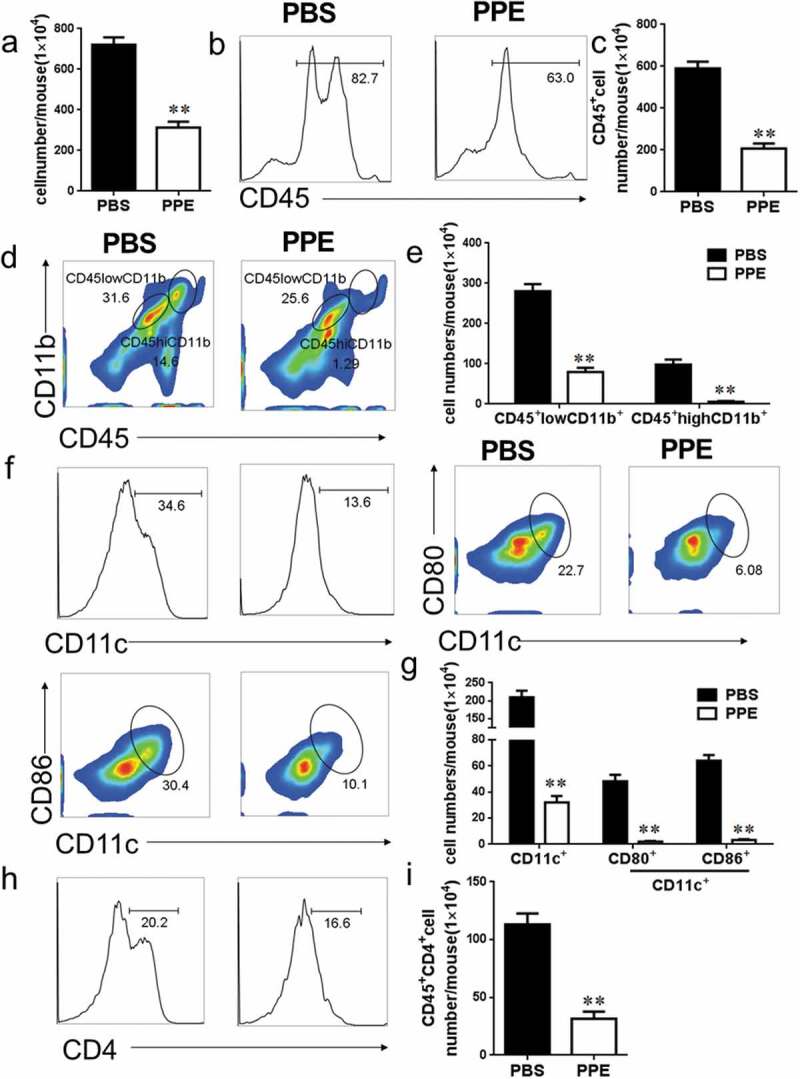
PPE treatment reduced CNS inflammation. EAE mice treated with PBS or PPE described in Figure 1(a) were sacrificed at d 24 p.i., and the brain and spinal cord were harvested. (a) Total number of mononuclear cells (MNCs) in CNS. (b–i) The percentages and absolute numbers of CD45^+^, CD45^+^CD11b^+^, CD11c^+^, CD11c^+^CD80^+^, CD11c^+^CD86^+^, CD4^+^ cells were measured by flow cytometry. Data are expressed as the mean ± SEM (n = 3 for each group). **p < 0.01, determined by unpaired Student’s *t*-test

**Figure 3.**
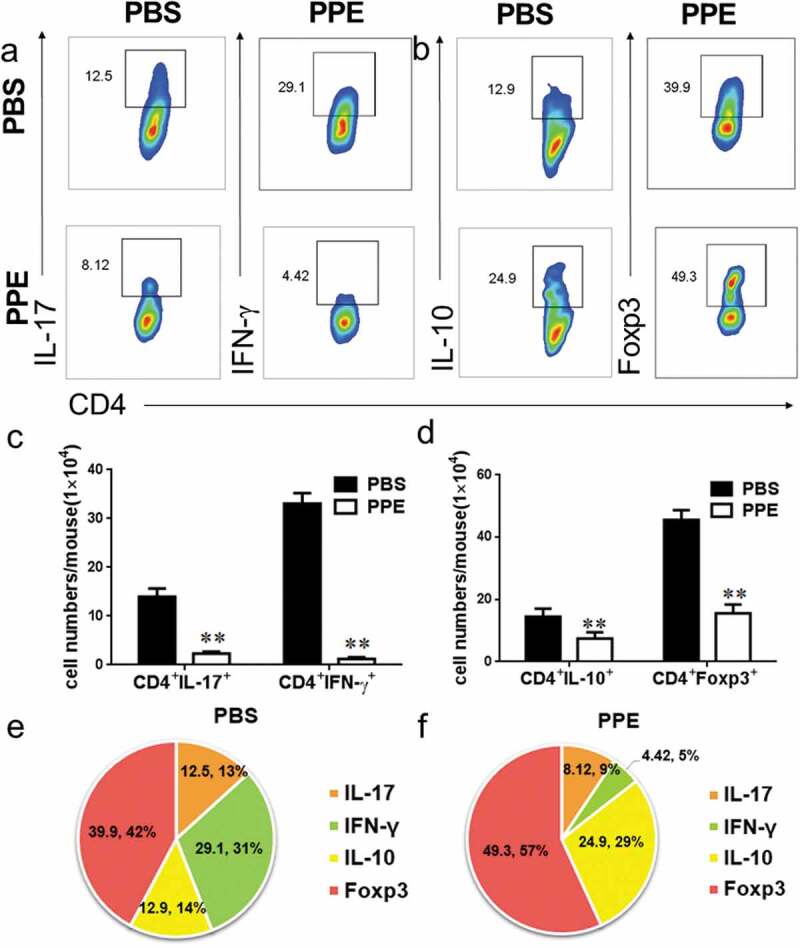
Effect of PPE treatment on T cell inflammatory factors in CNS. Tissue extraction and processing are the same as shown in Figure 2. (a–d) Assess the proportion and absolute numbers of IL-17^+^, IFN-γ^+^, IL-10^+^, and Foxp3^+^ cells in CD4^+^ cells by flow cytometry. (e, f) The percentage of these cells in the CD4^+^ cells in the CNS. Data are expressed as the mean ± SEM (n = 3 for each group). **p < 0.01, determined by unpaired Student’s *t*-test

Autoreactive T cells and monocytes migrate from the blood to the CNS, where they are further activated with microglia and CNS macrophages to cause inflammation and promote myelin destruction.^2^ Isolation of MNCs from CNS and analysis of the effects of PPE on CNS inflammation by flow cytometry. The total number of MNCs per mouse in the PBS-treated group and the PPE-treated group was 703.8 ± 119.0 × 10^4^, 382.6 ± 93.59 × 10^4^, respectively (*p* < 0.01, Figure 2(a)). The percentage and absolute number of infiltrating CD45^+^, CD11b^+^ macrophages, CD11b^+^ microglia, CD11b^+^, CD11c^+^, CD11c^+^CD80^+^, CD11c^+^CD86^+^ and CD4^+^ T cells in PPE-treated mouse CNS were significantly reduced compared to PBS-treated mice (*p* < 0.01, Figure 2(b–i)). Furthermore, it was observed that CD4^+^IL-17^+^, CD4^+^IFN-γ^+^ cells were less infiltrated in the CNS of PPE-treated mice (Figure 3(a)). Of note, the percentage of CD4^+^IL-10^+^, CD4^+^Foxp3^+^ cells increased after PPE treatment (Figure 3(b)). Since the total number of cells in the PBS control group was significantly higher than the total number of cells in the PPE treatment group, the absolute number of Treg cells did not increase during PPE treatment (*p* < 0.01, Figure 3(c, d)). However, compared with the PBS group, the results showed that the proportion of proinflammatory T cells in the PPE-treated group decreased, while the proportion of Treg cells increased (Figure 3(e, f)). These results showed that PPE significantly inhibited the infiltration of peripheral inflammatory cells into CNS.

### PPE modulated peripheral immune response

**Figure 4.**
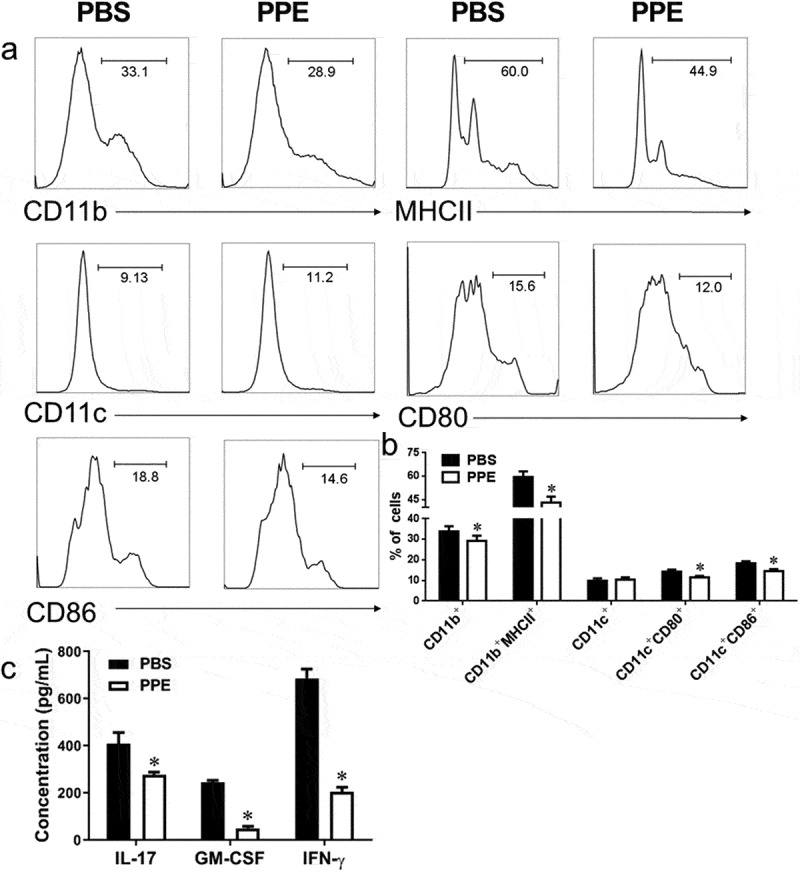
PPE treatment inhibited peripheral inflammation and pro-inflammatory cytokine production. As shown in Figure 1(a), mice were treated with PBS or PPE on the day of EAE immunization and sacrificed at d 24 p.i. to collect the spleen. Splenocytes were stimulated with 25 µg/mL MOG_35–55_ for 3 day. (a, b) The percentage and statistics of CD11b^+^, CD11b^+^MHCII^+^, CD11c^+^, CD11c^+^CD80^+^, CD11c^+^CD86^+^ were measured by flow cytometry. (c) Cytokine concentration in the cultured splenocyte supernatant was measured by ELISA. Data are expressed as the mean ± SEM (n = 3 for each group). *p < 0.05, determined by unpaired Student’s *t*-test

To assess the effect of oral PPE on the immune response, we used flow cytometry to study surface markers and/or cytokine expression in mononuclear cells from the peripheral. A significant reduction in the percentage of inflammatory cells was detected in the spleens of PPE-treated EAE mice compared to PBS control, including CD11b^+^, CD11b^+^MHCII^+^, CD11c^+^, CD11c^+^CD80^+^, CD11c^+^CD86^+^ activated DCs (*p*< 0.05, Figure 4(a, b)). CD4^+^ T cells, CD4^+^IL-17^+^, and CD4^+^IFN-γ^+^ Th17 cells also decreased in the PPE treatment group, but the difference was not significant (Supplementary Figure S2(a, b)). Notably, the percentage of Treg cells (CD4^+^Foxp3^+^, CD4^+^IL-4^+^, CD4^+^IL-10^+^) in the spleens of PPE-treated EAE mice was increased compared to PBS (*p*< 0.05, Supplementary Figure S2(c, d)). In addition, to examine the effect of PPE on MOG-stimulated cytokine secretion, the supernatant of spleen cells was analyzed by enzyme-linked immunoassay (ELISA), and the inflammatory cytokines IL-17, IFN-γ, and GM-CSF in the PPE-treated group were significantly reduced (*p*< 0.05, Figure 4(c)). These data indicated that PPE alleviated the severity of EAE by restraining DC activation and Th17 cell differentiation, inducing the production of immunoregulatory cytokines.

### Fecal microbiota transplantation (FMT) from PPE mice to naïve recipients ameliorates EAE clinical course

**Figure 5.**
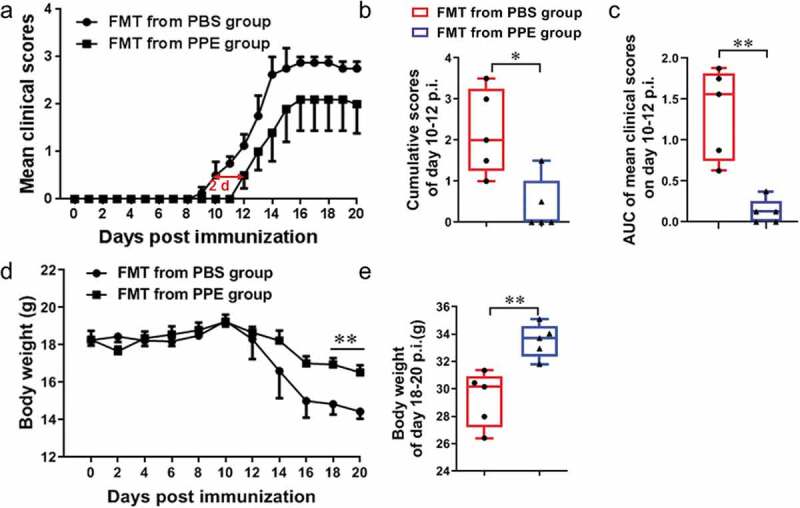
Fecal microbiota transplantation (FMT) from PPE-treated mice has a protective effect on EAE. Feces of EAE mice treated with PBS or PPE were collected, and perform FMT on recipient mice. FMT was administered daily from the day of immunization. (a) Clinical score and (d) body weight of EAE mice transferred with fecal matter from PPE or PBS group (n = 5). (b) Cumulative scores from d 10 to d 12 p.i. of the disease. (c) AUC of mean clinical scores on d 10–12 p.i. (e) Body weight of mice from d 18 to 20 p.i. Data are expressed as the mean ± SEM. *p < 0.05, **p < 0.01, determined by unpaired Student’s *t*-test. Data are represented by three independent experiments

FMT experiments were performed to investigate whether PPE improves EAE by modulating the gut microbiota. We transferred feces from mice on PPE or PBS group into 8-week-old naïve recipient mice by oral gavage, followed by immunization with MOG_35–55_ to induce EAE. Results shown that, compared to the recipients of FMT from PBS-feed mice, recipients of FMT from the PPE-treated mice delayed disease onset~2 d (Figure 5(a)). After induction of EAE, the cumulative scores of disease onset (d 10–12 p.i.) in the FMT from PBS group were 2.16, while the mice treated with FMT from PPE were reduced to 0.67 (*p*< 0.05, Figure 5(b)). Accordingly, the area under curve (AUC) of mean clinical scores from d 10 p.i. to d 12 p.i. of the PPE group was significantly lower than that of the PBS group (*p*< 0.01, Figure 5(c)). In addition, FMT from PPE group alleviated weight loss significantly, especially in the 18–20 d after immunization (*p*< 0.05, Figure 5(d, e)). Thus, improvement in EAE by PPE may be mediated, at least in part, by regulation in the gut microbiota.

### PPE treatment altered intestinal microbial composition in the EAE mice

**Figure 6.**
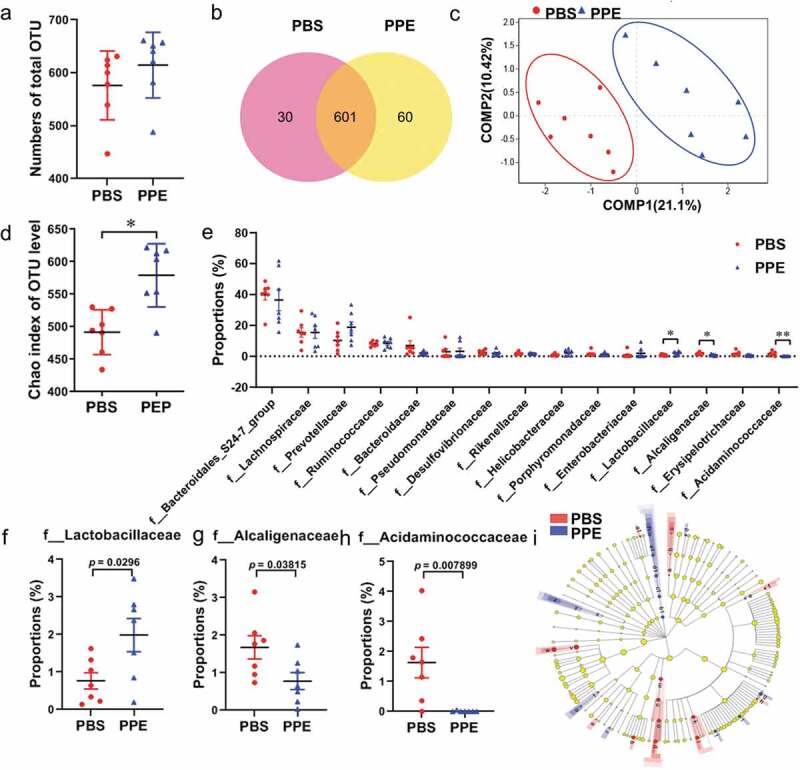
Comparison of gut microbiota characteristics of EAE mice treated with PBS and PPE. 16s rRNA sequencing were performed on stool samples from PBS and PPE treatment groups (n = 7). (a) Total OTU counts of gut microbiota in each group. (b) Venn diagram of the two groups. (c) Partial least-square discriminant analysis (PLS-DA) in PBS (red points) and PPE (blue points) group. Distance algorithm (Bray-Curtis) was used to calculate the significance of differences between two groups samples (*p*= 0.0001). (d) Chao index (α diversity) of gut microbiota at the OTU level. (e) Family level analysis of significant difference bacterial species between different groups. Comparison proportion of family levels of (f) Lactobacillaceae, (g) Alcaligenaceae and (h) Acidaminococcaceae. (i) Linear discriminant analysis effect size (LEfSe) was used to calculate the microbiota difference between the two groups. Determined by non-parametric factorial Kruskal-Wallis (KW) sum-rank test. Red and blue nodes indicate microbial groups that are significantly enriched in the PBS and PPE groups, and have a significant effect on the differences between groups; The yellow nodes represent that there is no significant difference between the two groups of microbiome, phylum, class, order, family, and genus are represented by circles from inside to outside. Abbreviation of the letters was shown in Supplementary Table S2. Data are expressed as the mean ± SEM. *p < 0.05, **p < 0.01, determined by Student’s *t*-test

Fecal samples were collected at the peak time point of the clinical disease. The V3-V4 hypervariable region sequencing of 16S rDNA was used to analyze the changes in the gut microbiota induced by PPE. No significant change was observed in the total OTU count of gut microbiota (Figure 6(a)). Venn diagram was used to visualize and compare commonalities and characteristics of species (such as OTU) in environmental samples. The common OTU of PPE and PBS was 601, and the characteristic OUT of PBS was 30, of which 60 OUT was unique to PPE (Figure 6(b)). Alpha diversity reflects the richness and diversity of microbiota diversity. Partial least squares discriminant analysis (PLS-DA) showed that there was a clear clustering pattern between PPE and PBS (Figure 6(c)). The Chao index (α-diversity) of gut microbiota increased significantly under PPE treatment (Figure 6(d)). Percent of community abundance on family level illustrated an alteration in the composition of the gut microbiota, the relative abundance of Prevotellaceae was higher and Bacteroidales_S24_7 was lower in the PPE group than that of PBS-treated mice (Supplementary Figure S3(a, b)). These data indicated that the composition of gut microbiota has been basically reshaped under PPE treatment.

Student’s *t*-test was used to analyze the differences between groups at the family level. The abundance of Lachnospiraceae, Prevotellaceae, Ruminococcaceae, Pseudomonadaceae, Helicobacteraceae, Enterobacteriaceae increased in the PPE treatment group (Figure 6(e)). Furthermore, the relative abundance of Lactobacillaceae was significantly increased (*p*< 0.05), while a dramatically lower abundance of Alcaligenaceae (*p*< 0.05), Acidaminococcaceae (*p*< 0.01) in PPE group compared with PBS group (figure 6(f–h)). In addition, linear discriminant analysis effect size (LEfSe) and linear discriminant analysis (LDA) were estimated to count gut microbiota that have a significant discriminate between the PBS- and PPE-treated EAE mice. The abundance of gut microbiota from phylum to genus levels was significantly different between the two groups. f__Bacteroidaceae, f__Peptostreptococcaceae, g__*Romboutsia*, c__Negativicutes: o__Selenomonadales, f__Acidaminococcaceae, g__*Phascolarctobacterium* and g__*Allobaculum* were more abundant in the PBS group, c__Deferribacteres, o__Deferribacterales, f__Deferribacteraceae, g__*Mucispirillum* and g__*Ruminococcaceae_UCG_013* were more abundant in the PPE group (Figure 6(i)). The larger the value of LDA, the greater the impact of species abundance on the difference between the two groups. LDA revealed that a higher abundance of Ruminococcaceae at the family level was noticed in the PPE group, whereas PBS group was dominated by Bacteroidaceae at the family level (Supplementary Figure S3(c)). In general, oral PPE had a profound impact on the composition and abundance of the gut microbiome.

## Discussion

MS is a complex disease with a high disability rate among young adults. Despite extensive research, the underlying causes and pathogenesis of MS remain opaque.^22^ Numerous studies have found natural products as a huge asset, showing excellent efficacy on MS in immune regulation, anti-inflammatory, and protection and repair of the central nervous system.^23^ PPE is the source of abundant natural phenolic compounds, including flavonoids, tannins, phenolic acids. Anti-inflammatory, antioxidant, anticancer, and antimutagenic activities are the main ways in which PPE promotes health.^12^ Few studies have treated the effects of PPE on autoimmune diseases, such as MS. Previous studies have shown that PPE can effectively down-regulated the clinical symptoms of EAE in DA rats,^24^ while the underlying mechanism is unknown. CD4^+^ T cells secreting IFN-γ and IL-17 are considered to be pathogens of MS, whereas regulatory T cells can suppress immune responses.^25^ Foxp3 is the essential transcriptional activator of Treg cells, which regulates the release of anti-inflammatory factors such as IL-10, thereby promoting partial remyelination and alleviating clinical symptoms.^2,26^ Microglia and macrophages are activated in MS and secrete pro-inflammatory cytokines.^27^ In addition, activated microglia and macrophages interact with encephalitogenic T cells via intercellular contact and cytokine-mediated communication, exacerbating neuroinflammation in EAE.^28^ Herein, we evidence that oral PPE daily can effectively reduce the clinical score, demyelination, and axonal damage in MOG_35-55_-induced EAE mice. In addition, PPE treatment inhibited DC/microglia/macrophages activation, reduced the production of Th17 cells, and raised the number of Tregs.

The low bioavailability and metabolism of polyphenols have been recognized after consuming pomegranate extracts.^29,30^ Unmodified polyphenols (90–95%) and conjugates are excreted into the intestinal lumen by bile, accumulated in the large intestine through the gastrointestinal tract at high concentrations, and then degraded by the gut microbiota into a large amount of low molecular weight compounds.^31^ Urolithins are the major metabolites of pomegranate derived from the tannin-gut microbiota interaction. Recently, Rocío García-Villalba et al. identified three new types of urolithin metabolites in human feces and urine.^32^ It is reported that urolithin mediates the neuroprotective effects of Alzheimer’s disease.^29^ The treatment of camu camu (*Myrciaria dubia*) rich in polyphenols prevents obesity by regulating the gut microbiota.^33^ Therefore, it is worthwhile to further investigate how dietary polyphenols can prevent and treat diseases by modulating the gut microbiota. FMT has been a great therapeutic potential for chronic gastrointestinal infections, inflammatory bowel disease, cardiometabolic, autoimmunity, and other extraintestinal diseases.^34^ Our study found that recipient mice receiving fecal transplantation from PPE mice also showed a remission of EAE, which supports the beneficial effects of PPE that can be partially mediated by gut microbiota. In addition, our study found that PPE treatment increased the abundance of gut microbiota in EAE mice, notably the relative abundance of Lactobacillaceae was significantly increased, with low relative abundance was found in the Alcaligenaceae, Acidaminococcaceae. Lactobacillaceae as a probiotic strain has been shown to reduce the severity of EAE.^9^ Several members of the Alcaligenaceae are suspected of humans and animals opportunistic pathogens, especially in children with autism.^35^ Li Ning et al. found a significant increase in the abundance of Alcaligenaceae in autism spectrum disorders-children's group.^36^ Acidaminococcaceae may be associated with diet-induced obesity, resveratrol and quercetin can significantly inhibit its relative abundance.^37^ Studies have shown that the abundance of Prevotella, Lachnospiraceae, Ruminococcaceae, *Lactobacillus* in patients with MS is usually lower than that of healthy controls.^38^ Our result groups showed an increase in the abundance of Prevotellaceae, Lachnospiraceae, Ruminococcaceae, Lactobacillaceae after PPE treatment. These results indicated that oral PPE can reshape and improve the gut microbiota of EAE mice.

**Figure 7.**
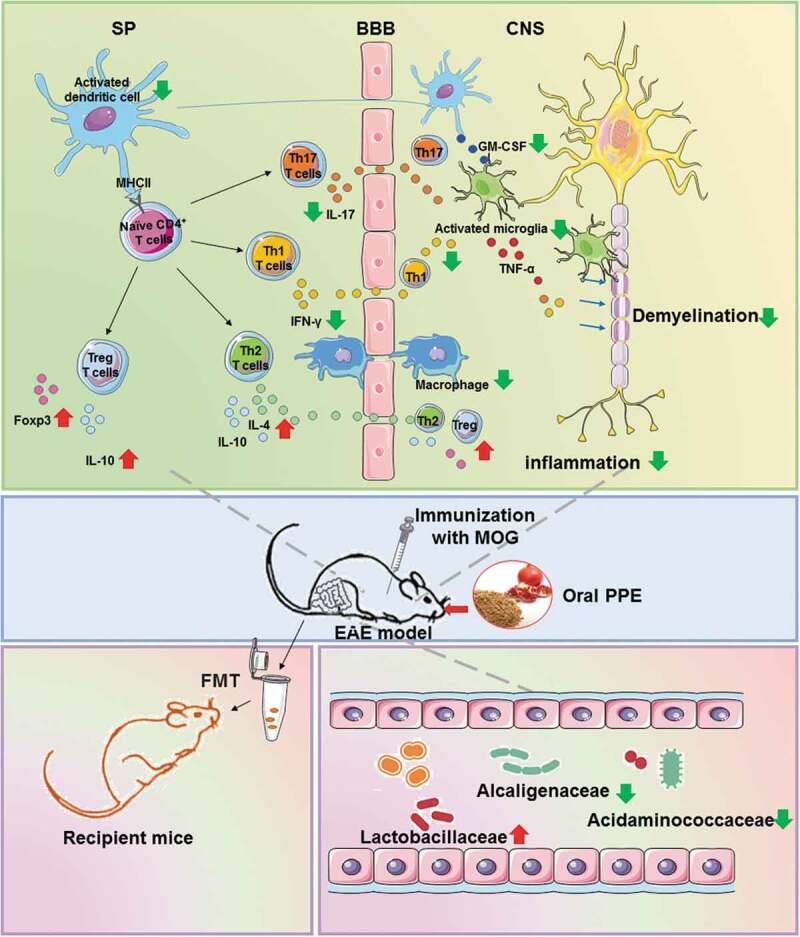
Health benefits of oral PPE on relieving the clinical symptoms of EAE and improving the gut microbiota

In conclusion, this study described the metabolite profiling of PPE, explored the therapeutic effects and action mechanism of PPE on EAE. We demonstrated that oral PPE had a positive preventive and therapeutic effect on EAE, effectively alleviated the deterioration of the disease, and inhibited neuroinflammation and demyelination. In addition, EAE mice treated with FMT from PPE group showed a significant delay of disease onset and obvious remission of the severity of the disease. The 16s rRNA sequencing results showed that the gut microbiota of PPE-fed mice was remodeled, with a significant increase in Lactobacillaceae, and a lower abundance of the Alcaligenaceae and Acidaminococcaceae (Figure 7). Our data indicated that PPE can effectively alleviate the clinical symptoms of EAE, inhibit the production of pro-inflammatory cytokines, and increase the expression of anti-inflammatory cytokines by altering the gut microbiota. Notably, our results provide a theoretical basis for the deep development and utilization of pomegranate resources.

## Supplementary Material

Supplemental Material

## Notes

The author claims that there is no competitive interest.
