# Supplementary material for: Pomegranate peel extract ameliorates the severity of experimental autoimmune encephalomyelitis via modulation of gut microbiota
Source: Gut Microbes. 2020 Dec 17;12(1):1857515. doi: 10.1080/19490976.2020.1857515 (PMC7751635; doi:10.1080/19490976.2020.1857515)
Supplement: Supplemental Material [file KGMI_A_1857515_SM0729.zip › Supplementary information/Supplemental Material legends.docx]

**Supplemental Material**

Figure S1. Metabolite profiling of pomegranate peel extracts (PPE). (A) HPLC chromatogram of ellagic acid standard. (B) HPLC chromatogram of PPE sample. (C) Typical GC−MS total ion chromatograph of PPE. (D) Percentage of chemical components in PPE.

Figure S2. Effect of PPE treatment on T cell inflammatory factors in the periphery. Tissue extraction and processing are the same as shown in Figure 4. (A-D) The percentage and statistics of IL17^+^, IFN-γ^+^,IL-4^+^, IL-10^+^, Foxp3^+^ in CD4^+^ T cells were measured by flow cytometry. Data are expressed as the mean ± SEM (n = 3 for each group). **p* < 0.05, determined by unpaired Student’s t-test.

Figure S2. Oral PPE regulates the gut microbiota diversity of EAE mice (n = 7). (A-B) Community analysis pie plot on family level in the gut microbiota of two groups. (C) The LDA was analyzed and the LDA score > 2.5 was displayed.

Table S1. Detail information of chemical composition of pomegranate peel extract determined by GC-MS.

Table S2. Abbreviation of LEfSe analysis.
