# Supplementary material for: Pomegranate peel extract ameliorates the severity of experimental autoimmune encephalomyelitis via modulation of gut microbiota
Source: Gut Microbes. 2020 Dec 17;12(1):1857515. doi: 10.1080/19490976.2020.1857515 (PMC7751635; doi:10.1080/19490976.2020.1857515)
Supplement: Supplemental Material [file KGMI_A_1857515_SM0729.zip › Supplementary information/Supplementary Table 1.pdf]

Supplementary Table 1: Detailed information of chemical composition of pomegranate peel extract determined by GC-MS.

ID	Name	RT_Min	Score	HMDBID	KeggID	Class
1	Pyruvic acid	5.29	852	HMDB00243	C00022	Organic Acids
2	L-Lactic acid	5.42	949	HMDB00190	C00186	Organic Acids
3	Phenol	5.43	878	HMDB00228	C00146	Phenols
4	Glycolic acid	5.62	919	HMDB00115	C00160	Organic Acids
5	L-Alanine	5.97	947	HMDB00161	C00041	Amino Acid
6	Oxalic acid	6.32	898	HMDB02329	C00209	Organic Acids
7	2_Furoic_acid	6.41	875	HMDB00617	C01546	Organic Acids
8	Methylmalonic acid	7.31	883	HMDB00202	C02170	Organic Acids
9	L-Valine	7.38	963	HMDB00883	C00183	Amino Acid
10	Urea	7.7	954	HMDB00294	C00086	Amino Acid
11	Benzoic acid	7.84	892	HMDB01870	C00180	Organic Acids
12	Caprylic acid	8	893	HMDB00482	C06423	Fatty Acids
13	Ethanolamine	8.01	973	HMDB00149	C00189	Alkylamines
14	DL_O_Phosphoserine	8.04	774	HMDB01721	C01005	Amino Acid
15	Phosphoric acid	8.04	982	HMDB02142	C00009	Phosphate
16	Glycerol	8.05	989	HMDB00131	C00116	Alcohols
17	L-Proline	8.39	903	HMDB00162	C00148	Amino Acid
18	Glycine	8.48	975	HMDB00123	C00037	Amino Acid
19	Succinic acid	8.56	982	HMDB00254	C00042	Organic Acids
21	Glyceric acid	8.71	957	HMDB00139	C00258	Organic Acids
20	Picolinic acid	8.71	835	HMDB02243	C10164	Organic Acids
22	Uracil	8.84	928	HMDB00300	C00106	Nucleotide
23	Citraconic_acid	8.96	879	HMDB00634	C02226	Organic Acids
24	Fumaric acid	8.98	919	HMDB00134	C00122	Organic Acids
25	L-Serine	9.07	983	HMDB00187	C00065	Amino Acid
26	L-Threonine	9.35	958	HMDB00167	C00188	Amino Acid
27	Beta-Alanine	9.86	905	HMDB00056	C00099	Amino Acid
28	Benzamide	9.98	828	HMDB04461	C09815	Alkylamines
29	Oxalacetic_acid	10.15	934	HMDB00223	C00036	Organic Acids
30	Capric acid	10.2	839	HMDB00511	C01571	Fatty Acids
31	2_Phenylacetamide	10.25	856	HMDB10715	C02505	Alkylamines
32	L-Malic acid	10.47	997	HMDB00156	C00149	Organic Acids

33	D-Threitol	10.57	939	HMDB04136	C16884	Carbohydrates
34	L-Aspartic acid	10.81	963	HMDB00191	C00049	Amino Acid
35	Pyrrolidonecarboxylic_acid	10.86	991	HMDB00805	C02237	NA
36	Gamma-Aminobutyric acid	10.95	992	HMDB00112	C00334	Amino Acid
37	Threonic acid	11.18	988	HMDB00943	C01620	Carbohydrates
38	Oxoglutaric acid	11.37	832	HMDB00208	C00026	Organic Acids
39	L-Glutamic acid	11.82	872	HMDB00148	C00025	Amino Acid
40	L-Phenylalanine	11.92	813	HMDB00159	C00079	Amino Acid
41	D-Xylose	12.03	896	HMDB00098	C00181	Carbohydrates
42	Dodecanoic acid	12.19	838	HMDB00638	C02679	Fatty Acids
43	L-Arabinose	12.19	983	HMDB00646	C00259	Carbohydrates
44	L-Asparagine	12.3	961	HMDB00168	C00152	Amino Acid
45	Taurine	12.3	826	HMDB00251	C00245	Organic Acids
46	D-Ribose	12.34	945	HMDB00283	C00121	Carbohydrates
47	D-Xylitol	12.61	863	HMDB02917	C00379	Carbohydrates
49	D_Arabitol	12.74	830	HMDB00568	C01904	Carbohydrates
48	L-Arabitol	12.74	992	HMDB01851	C00532	Carbohydrates
50	Rhamnose	12.78	917	HMDB00849	C00507	Carbohydrates
51	Ribitol	12.79	840	HMDB00508	C00474	Carbohydrates
52	Aminocaproic_acid	12.85	886	HMDB01901	C02378	NA
53	L-Fucose	12.94	814	HMDB00174	C01019	Carbohydrates
54	cis_Aconitic_acid	13.03	863	HMDB00072	C00417	Organic Acids
55	Glycerol 3-phosphate	13.12	908	HMDB00126	C00093	Lipids
56	Vanillic_acid	13.22	863	HMDB00484	C06672	Organic Acids
57	L-Glutamine	13.27	830	HMDB00641	C00064	Amino Acid
58	p_Hydroxymandelic_acid	13.35	882	HMDB00822	C11527	NA
59	Shikimic acid	13.56	966	HMDB03070	C00493	Organic Acids
60	Citric acid	13.66	978	HMDB00094	C00158	Organic Acids
61	Isocitric acid	13.66	897	HMDB00193	C00311	Organic Acids
62	Myristic acid	13.98	885	HMDB00806	C06424	Fatty Acids
63	Quinic acid	14	917	HMDB03072	C06746	Organic Acids
64	L-Sorbose	14.11	991	HMDB01266	C08356	Carbohydrates
65	D-Fructose	14.19	977	HMDB00660	C02336	Carbohydrates
66	Gluconolactone	14.23	906	HMDB00150	C00198	Carbohydrates

67	D-Mannose	14.34	928	HMDB00169	C00159	Carbohydrates
69	D-Glucose	14.5	906	HMDB00122	C00031	Carbohydrates
70	D-Galactose	14.5	994	HMDB00143	C00984	Carbohydrates
72	Sorbitol	14.62	740	HMDB00247	C00794	Carbohydrates
71	Mannitol	14.62	956	HMDB00765	C00392	Carbohydrates
73	Ascorbic acid	14.77	828	HMDB00044	C00072	Vitamin
74	Indoleacetic acid	14.98	892	HMDB00197	C00954	Indoles
75	Pantothenic acid	15.15	825	HMDB00210	C00864	Vitamin
76	Gluconic acid	15.19	943	HMDB00625	C00257	Carbohydrates
77	Guanidinosuccinic acid	15.34	921	HMDB03157	C03139	Organic Acids
78	Palmitic acid	15.62	970	HMDB00220	C00249	Fatty Acids
79	N_Acetyl_D_glucosamine	15.82	870	HMDB00215	C00140	Carbohydrates
80	Myoinositol2	15.94	992	HMDB00211	C00137	Alcohols
81	Myoinositol1	15.94	839	HMDB02256	C00137	NA
82	Uric acid	15.98	920	HMDB00289	C00366	Organic Acids
83	Normetanephrine	16.13	893	HMDB00819	C05589	Hormone
84	Heptadecanoic acid	16.39	837	HMDB02259	NA	Fatty Acids
85	Indolelactic acid	16.62	941	HMDB00671	C02043	Indoles
86	Linoleic acid	16.9	957	HMDB00673	C01595	Fatty Acids
88	Oleic acid	16.94	815	HMDB00207	C00712	Fatty Acids
87	Petroselinic acid	16.94	877	HMDB02080	C08363	Organic Acids
89	Stearic acid	17.13	945	HMDB00827	C01530	Fatty Acids
90	Arachidic acid	18.52	864	HMDB02212	C06425	Fatty Acids
91	Sucrose	19.72	991	HMDB00258	C00089	Carbohydrates
92	Adenosine	19.75	858	HMDB00050	C00212	Nucleotide
93	Androstenedione	19.77	883	HMDB00053	C00280	Lipids
94	Behenic acid	19.8	896	HMDB00944	C08281	Fatty Acids
95	Alpha-Lactose	20.04	871	HMDB00186	C00243	Carbohydrates
96	Cellobiose	20.28	822	HMDB00055	C06422	Carbohydrates
97	D-Maltose	20.51	923	HMDB00163	C00208	Carbohydrates
98	Guanosine	20.55	868	HMDB00133	C00387	Nucleotide
99	Maltitol	20.98	831	HMDB02928	NA	Carbohydrates
100	Tetracosanoic acid	21.05	832	HMDB02003	C08320	Fatty Acids
101	Genistein	21.87	929	HMDB03217	C06563	Flavonoids
