# Supplementary material for: Pomegranate peel extract ameliorates the severity of experimental autoimmune encephalomyelitis via modulation of gut microbiota
Source: Gut Microbes. 2020 Dec 17;12(1):1857515. doi: 10.1080/19490976.2020.1857515 (PMC7751635; doi:10.1080/19490976.2020.1857515)
Supplement: Supplemental Material [file KGMI_A_1857515_SM0729.zip › Supplementary information/Supplementary Table 2.pdf]

Supplementary Table 2: Abbreviation of LEfSe analysis.

Letter	Taxa
a	o__Pasteurellales
b	f__Pasteurellaceae
c	g__Pasteurella
d	g__unclassified_f__Ruminococcaceae
e	g__Ruminococcaceae_UCG_013
f	g__Ruminococcaceae_NK4A214_group
g	f__Peptostreptococcaceae
h	g__Romboutsi
i	c__Negativicutes
j	o__Selenomonadales
k	f__Acidaminococcaceae
l	g__Phascolarctobacterium
m	g__Allobaculum
n	g__Lactococcus
o	f__Bacteroidaceae
p	g__Bacteroides
q	o__Solibacterales
r	f__Solibacteraceae__Subgroup_3_
s	g__Candidatus_Solibacter
t	g__Gordonibacter
u	p__Deferribacteres
v	c__Deferribacteres
w	o__Deferribacterales
x	f__Deferribacteraceae
y	g__Mucispirillum
