# Supplementary figures and images for: Pomegranate peel extract ameliorates the severity of experimental autoimmune encephalomyelitis via modulation of gut microbiota
Source: Gut Microbes. 2020 Dec 17;12(1):1857515. doi: 10.1080/19490976.2020.1857515 (PMC7751635; doi:10.1080/19490976.2020.1857515)

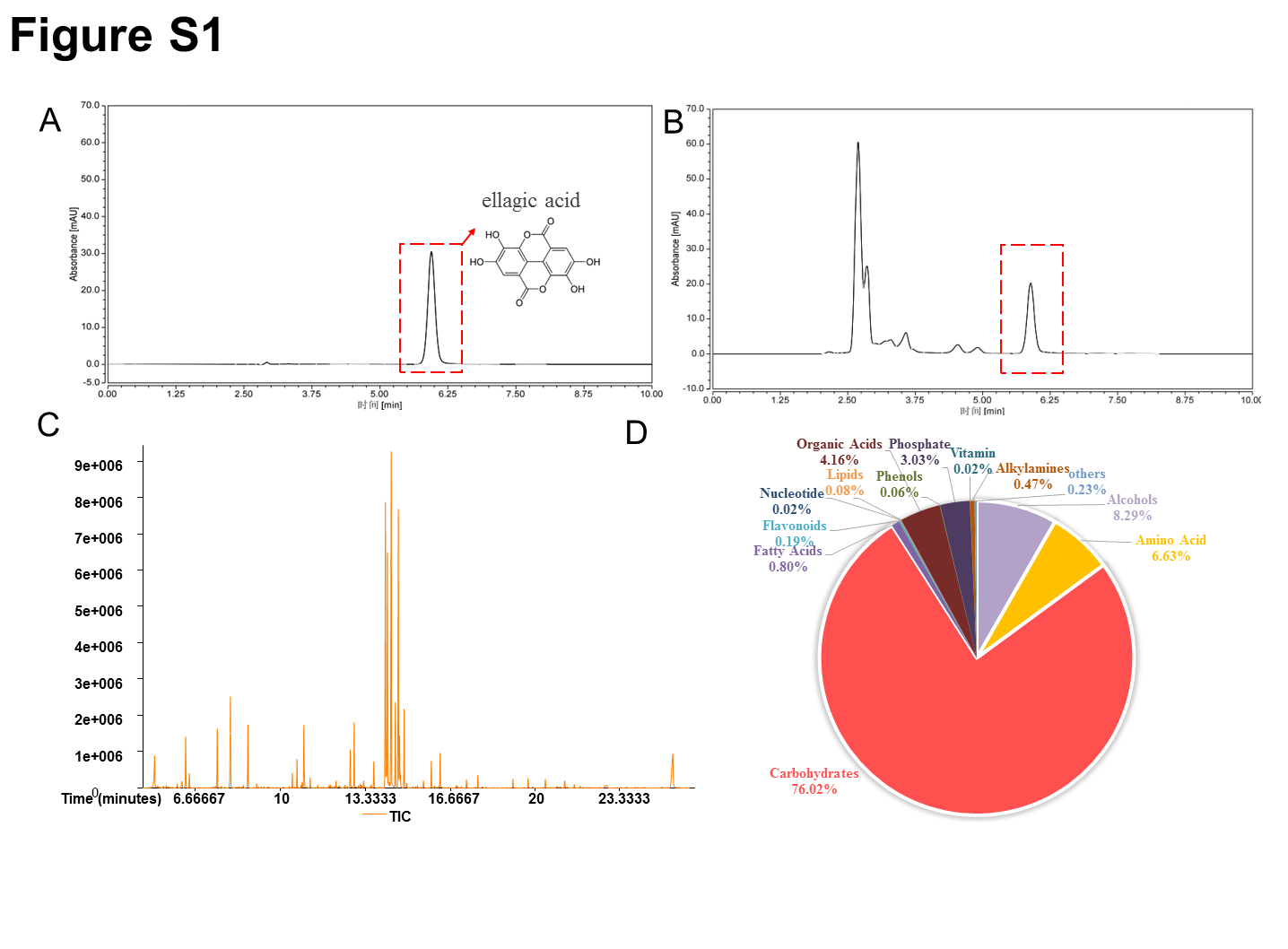

Supplement: Supplemental Material [file KGMI_A_1857515_SM0729.zip › Supplementary information/Figure S1.tif]

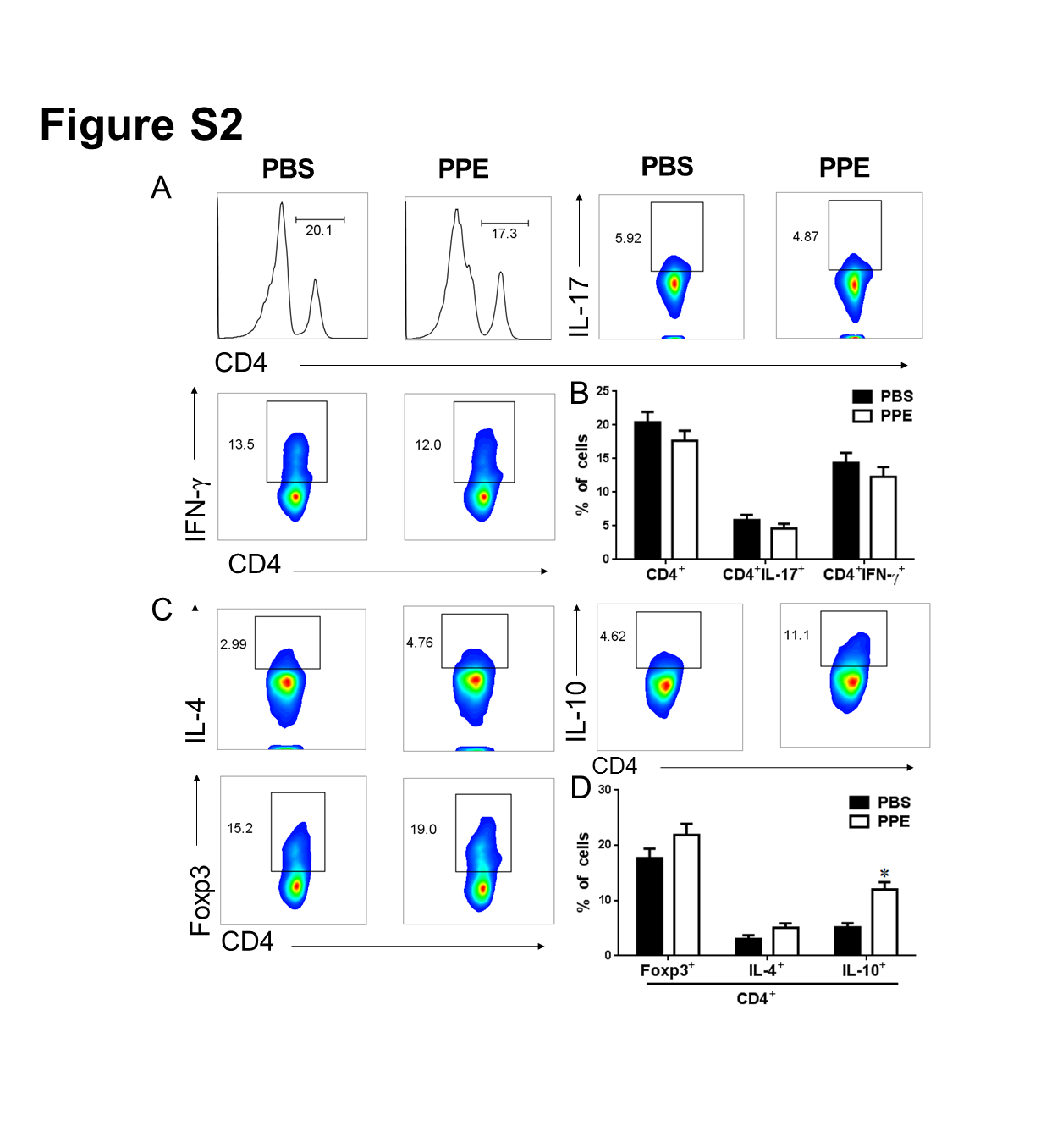

Supplement: Supplemental Material [file KGMI_A_1857515_SM0729.zip › Supplementary information/Figure S2.TIF]

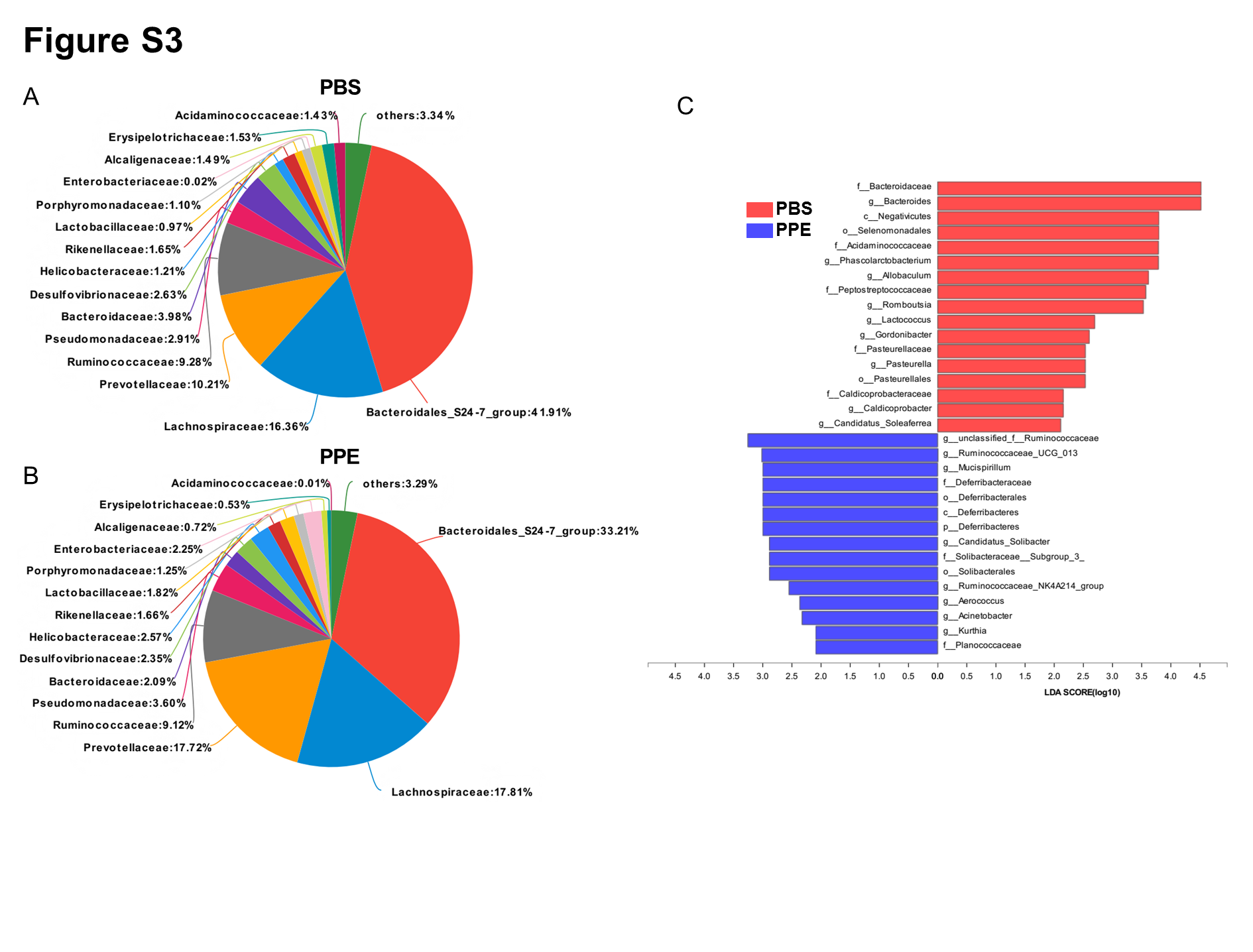

Supplement: Supplemental Material [file KGMI_A_1857515_SM0729.zip › Supplementary information/Figure S3.tif]
